# Influence of antibody–drug conjugate cleavability, drug-to-antibody ratio, and free payload concentration on systemic toxicities: A systematic review and meta-analysis

**DOI:** 10.1007/s10555-024-10231-5

**Published:** 2024-12-20

**Authors:** Shou-Ching Tang, Carrie Wynn, Tran Le, Martin McCandless, Yunxi Zhang, Ritesh Patel, Nita Maihle, William Hillegass

**Affiliations:** 1 LSU-LCMC Cancer Center, Louisiana State University (LSU) Health Sciences Center, New Orleans, LA U.S.A.; 2 Department of Medicine, University of Mississippi Medical Center, Jackson, MS U.S.A.; 3 Department of Data Science, University of Mississippi Medical Center, Jackson, MS U.S.A.; 4 Department of Neurological Surgery, Medical Center, University of Kansas, Kansas City, KS U.S.A.; 5 Trinity Health Ann Arbor Hospital, Ann Arbor, MI U.S.A.; 6 Departments of Data Science and Medicine, University of Mississippi Medical Center, Jackson, MS U.S.A.

**Keywords:** Antibody–drug conjugates, Cleavable and non-cleavable linkers, Payloads, Drug to antibody ratio, Systemic toxicities, Meta-analysis

## Abstract

While in theory antibody drug conjugates (ADCs) deliver high-dose chemotherapy directly to target cells, numerous side effects are observed in clinical practice. We sought to determine the effect of linker design (cleavable versus non-cleavable), drug-to-antibody ratio (DAR), and free payload concentration on systemic toxicity. Two systematic reviews were performed via PubMed search of clinical trials published between January 1998—July 2022. Eligible studies: (1) clinical trial for cancer therapy in adults, (2) ≥ 1 study arm included a single-agent ADC, (3) ADC used was commercially available/FDA-approved. Data was extracted and pooled using generalized linear mixed effects logistic models. 40 clinical trials involving 7,879 patients from 11 ADCs, including 9 ADCs with cleavable linkers (*N* = 2,985) and 2 with non-cleavable linkers (*N* = 4,894), were included. Significantly more composite adverse events (AEs) ≥ grade 3 occurred in patients in the cleavable linkers arm (47%) compared with the non-cleavable arm (34%). When adjusted for DAR, for grade ≥ 3 toxicities, non-cleavable linkers remained independently associated with lower toxicity for any AE (*p* = 0.002). Higher DAR was significantly associated with higher probability of grade ≥ 3 toxicity for any AE. There was also a significant interaction between cleavability status and DAR for any AE (*p* = 0.002). Finally, higher measured systemic free payload concentrations were significantly associated with higher DARs (*p* = 0.043). Our results support the hypothesis that ADCs with cleavable linkers result in premature payload release, leading to increased systemic free payload concentrations and associated toxicities. This may help to inform future ADC design and rational clinical application.

## Introduction

Antibody–drug conjugates (ADCs) are monoclonal antibodies connected to a cytotoxic agent known as the payload via a chemical linker. It was hoped that ADCs would be “magic bullets,” delivering high-dose cytotoxic chemotherapy directly to cancer cells without affecting surrounding normal tissues. However, this has not borne out in clinical practice. Though many factors affect toxicity, the toxicities of currently approved ADCs appear to be driven primarily by premature release of the payload into the bloodstream by the linker, by an excessively prominent bystander effect [1], or even payload released by the lysed tumor cells [2].

ADC linkers can be divided broadly into two groups: cleavable and non-cleavable. Cleavable linkers such as hydrazone, disulfide, or peptide linkers rely on physiologic factors (i.e., cathepsin, glutathione (GSH), and low pH) within the cell to cleave the linker. Because these conditions can occur independently of antigen internalization, cleavable linkers are often less stable in the blood, resulting in various off-target effects [3]. In contrast, non-cleavable linkers, such as the thioether or maleimidocaproyl linkers, require internalization by the target cell, so that the antibody, rather than the linker, can be degraded by the lysosome before the drug is released. This latter mechanism does not produce efficient bystander killing and thus results in lower toxicity profiles [4]. For these reasons, other novel linkers, including conditionally released linkers, are currently in rapid development [5, 6].

Preclinical studies have shown that compared to ADCs with non-cleavable linkers, those with cleavable linkers likely release free payload prematurely, leading to increased systemic toxicity. In this study, we sought to delineate the potential effect of linker design on systemic toxicity by analyzing the results of clinical trials using ADCs constructed with both types of linkers. We hypothesized that ADCs with cleavable linkers would be associated with greater systemic toxicities than those with non-cleavable linkers. To test this hypothesis, we conducted a systematic review of adverse events (AEs) occurring in cancer patients treated with commercially available ADCs. We then carried out a meta-analysis on all eligible phase II-III clinical trials. We also evaluated the potential effect of drug-to-antibody ratio (DAR) and systemic free payload concentration on toxicity in the context of the cleavability of the linkers used.

## Materials and methods

### Search methods and study selection

Following the Preferred Reporting Items for Systematic Reviews and Meta-analyses (PRISMA) 2020 updated guidance, two systematic reviews were performed through a PubMed search on July 5, 2022. The first identified studies reporting clinical toxicity rates. Studies eligible for inclusion met the following criteria: (1) clinical trial for cancer therapy in adults ages 18 or older, (2) participants in at least one arm of the study were treated with single-agent ADC, (3) the ADC used was commercially available and FDA-approved for cancer treatment as of July 5, 2022, (4) the study reported treatment-related adverse events, and (5) the study was published in English. Studies where the ADC was administered in combination with other chemotherapy agents were excluded because the ADC’s individual contribution to the overall toxicity profile of the regimen could not be fully delineated. A second systematic review identified studies reporting the DARs and estimated systemic free payload concentrations for all FDA-approved ADCs.

### Statistical analysis: meta-analysis of clinical toxicity rates

To compare the incidence of toxicities in patients treated with ADCs constructed with cleavable vs non-cleavable linkers, generalized linear mixed effects logistic models were conducted for each toxicity, including:

1. *By linker type*. Univariable mixed effects logistic regression models were constructed evaluating the association between frequency of each specific toxicity for the binary outcome variables (any grade vs none; grade ≥ 3 versus grade ≤ 2) and ADC linker type (cleavable vs non-cleavable).

2. *Linker type adjusted for drug-to-antibody ratio*. Multivariable mixed effects logistic regression models were constructed evaluating the association between frequency of each specific toxicity for the binary outcome variables (any grade vs none; grade ≥ 3 vs grade ≤ 2) and ADC linker type (cleavable vs non-cleavable) plus drug-to-antibody ratio (numeric predictor) with the potential interaction between ADC linker type and drug-to-antibody ratio when estimable. The model includes the interaction when estimable since cleavability status and drug-to-antibody ratio are not necessarily independent factors but arise from the design of each medication.

3. *Linker type adjusted for estimated systemic free payload concentration*. Multivariable mixed effects logistic regression models were constructed evaluating the association between frequency of each specific toxicity for the binary outcome variables (any grade vs none; grade ≥ 3 vs grade ≤ 2) and ADC linker type (cleavable vs non-cleavable) plus systemic free payload concentration (numeric predictor) with the potential interaction between ADC linker type and payload systemic free concentration when estimable. Again, the model includes the interaction when estimable since cleavability status and systemic free payload concentration are not necessarily independent factors but arise from the design of each medication.

Heterogeneity in the estimated probability of each specific toxicity between studies was assessed with the I [2] statistic, describing the percentage of variation in probability of each specific toxicity across the studies arising from differences in the included trials (heterogeneity) rather than sampling error (chance).

## Results

### Study inclusion

**Fig. 1:**
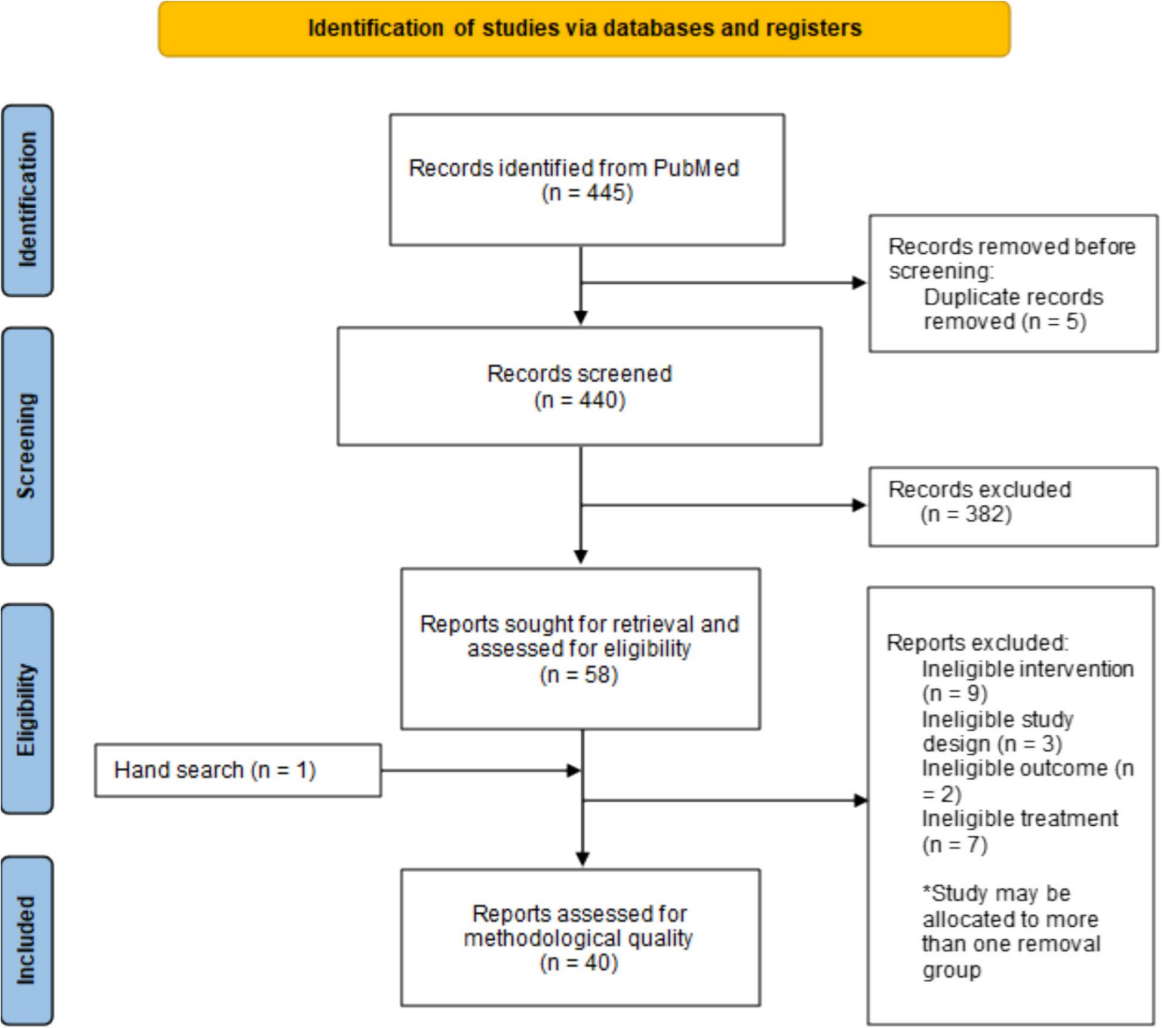
PRISMA flowchart describing the result of the search and selection process

A literature search and review of references identified 440 relevant publications after duplicates were removed. After eligibility assessment, a total of 40 clinical trials involving 7,879 patients were used to perform our meta-analysis, as shown in Fig. 1 [7–46]. Eleven (11) commercially-available FDA-approved ADCs were included (Table 1). Nine of these studies reported the results of treatment with ADCs with cleavable linkers (*N* = 2,985), whereas two used non-cleavable linkers (*N* = 4,894). Table 2 lists the ADC agent, target disease, study design, number of patients treated with the ADC, and modified Newcastle–Ottawa scale study quality rating. Table 3 lists the 21 specific toxicities examined. It also indicates the number of included studies reporting the specific toxicity, patients at risk, and the number of patients experiencing toxicities for grade ≥ 3 (Table 3) and any grade (Table 4).

**Table 1:** Included ADCs with linker type, drug-to-antibody ratio, and systemic maximum free payload concentration

Antibody drug conjugate	Indication(s)	Antigen	Payload agent	Number of studies	Number of patients	Linker	Drug-to-antibody ratio (DAR)	Systemic maximum free payload concentration (kg/L)
Belantamab mafodotin	Multiple myeloma	BCMA	MMAF	1	97	Non-cleavable	4	0.0004
Brentuximab vedotin	Hodgkin lymphoma, anaplastic large cell lymphoma, CD30 + peripheral T cell lymphoma, CD30 + mycosis fungoides	CD30	MMAE	11	660	Cleavable	4	0.0026
Enfortumab vedotin	Urothelial cancer	Nectin-4	MMAE	3	515	Cleavable	3.8	0.0040
Gemtuzumab ozogamicin	Acute myeloid leukemia	CD33	Calichea-micin	2	175	Cleavable	2.5	0.0229
Inotuzumab ozogamicin	Acute lymphoblastic leukemia	CD22	Calichea-micin	2	205	Cleavable	6	0.0556
Loncastuximab tesirine	Large B-cell lymphoma	CD19	PBD dimer	1	145	Cleavable	2.3	0.0003
Moxetumomab pasudotox	Hairy cell leukemia	CD22	PE38	1	80	Cleavable	1	*No systemic accumulation observed*
Sacituzumab govitecan	Breast cancer Urothelial cancer	Trop-2	SN38	3	479	Cleavable	7.6	0.0120
Tisotumab vedotin	Cervical cancer	Tissue factor	MMAE	2	156	Cleavable	4	0.0026
Trastuzumab deruxtecan	Breast cancer Gastric cancer	HER2	Deruxtecan	3	570	Cleavable	8	0.4347
Trastuzumab emtansine	Breast cancer	HER2	Emtansine	12	4,797	Non-cleavable	3.5	0.0012

*BCMA* B-cell maturation antigen, *MMAE* monomethyl auristatin, *MMAF* monomethyl auristatin F, *PBD* pyrrolobenzodiazepine, *PE38* the 38 kDa fragment of Pseudomonas exotoxin A, *SN38* active metabolite of irinotecan

**Table 2:** Studies included in meta-analysis

ADC name	Study first author, year	Cancer type	Study design/phase/site	ADC group sample size	Modified NOS rating
Belantamab mafadotin	Lonial, 2021	Multiple myeloma	RCT/2/multicenter	97	Good
Brentuximab vedotin	Pro, 2012	Systemic ALCL	Single-arm study/2/Multicenter	58	Fair
	Younes, 2012	Hodgkin lymphoma	Single-arm study/2/Multicenter	102	Good
	Horwitz, 2014	PTCL	Single-arm study/2/Multicenter	35	Fair
	Monjanel, 2014	Hodgkin lymphoma, ALCL	Retrospective cohort study/2/Single center	45	Good
	Duvic, 2015	pcALCL, MF	Single-arm study/2/Single center	54	Good
	Kim, 2015	MF, Sezary syndromeS	Single-arm study/2/Multicenter	32	Fair
	Prince, 2017	MF, pcALCL	RCT/3/Multicenter	64	Good
	Walewski, 2018	Hodgkin lymphoma	Single-arm study/4/Multicenter	60	Good
	Stefoni, 2020	Hodgkin lymphoma	Single-arm study/2/Multicenter	18	Good
	Kuruvilla, 2021	Hodgkin lymphoma	RCT/3/Multicenter	153	Good
	Song, 2021	Hodgkin lymphoma, ALCL	Single-arm study/2/Multicenter	39	Fair
Enfortumab vedotin	Rosenberg, 2019	Urothelial cancer	Single-arm study/2/Multicenter	125	Good
	Powles, 2021	Urothelial cancer	RCT/3/Multicenter	301	Good
	Yu, 2021	Urothelial cancer	Single-arm study/2/Multicenter	89	Good
Gemtuzumab ozogamicin	Taksin, 2007	AML	Single-arm study/2/Multicenter	57	Fair
	Amadori, 2016	AML	RCT/3/Multicenter	118	Good
Inotuzumab ozogamicin	Kantarjian, 2013	ALL	Single-arm study/1.5/Single center	41	Good
	Kantarjian, 2019	ALL	RCT/3/Multicenter	164	Good
Loncastuximab tesirine	Caimi, 2021	DLBCL	Single-arm study/2/Multicenter	145	Good
Moxetumomab pasudoxtox	Kreitman, 2021	Hairy cell leukemia	Single-arm study/2/Multicenter	80	Good
Sacituzumab govitecan	Bardia, 2019	Breast cancer	Single-arm study/1.5/Multicenter	108	Good
	Bardia, 2021	Breast cancer	RCT/3/Multicenter	258	Good
	Tagawa, 2021	Urothelial cancer	Non-randomized multicohort study/2/Multicenter	113	Good
Tisotumab vedotin	Hong, 2020	Cervical cancer	Single-arm study/1.5/Multicenter	55	Fair
	Coleman, 2021	Cervical cancer	Single-arm study/2/Multicenter	101	Good
Trastuzumab deruxtecan	Modi, 2020	Breast cancer	Single-arm study/2/Multicenter	184	Good
	Shitara, 2020	Gastric cancer	RCT/2/Multicenter	125	Good
	Cortés, 2022	Breast cancer	RCT/3/Multicenter	261	Good
Trastuzumab emtansine	Burris, 2011	Breast cancer	Single-arm study/2/Multicenter	112	Good
	Krop, 2012	Breast cancer	Single-arm study/2/Multicenter	110	Good
	Verma, 2012	Breast cancer	RCT/3/Multicenter	495	Good
	Yardley, 2015	Breast cancer	Single-arm study/4/Multicenter	215	Good
	Kashiwaba, 2016	Breast cancer	Single-arm study/2/Multicenter	73	Good
	Krop, 2017	Breast cancer	RCT/3/Multicenter	403	Good
	Watanabe, 2017	Breast cancer	Single-arm study/2/Multicenter	232	Fair
	Montemurro, 2019	Breast cancer	Single-arm study/3b/Multicenter	2002	Good
	Von Minckwitz, 2019	Breast cancer	RCT/3/Multicenter	743	Good
	Cortés, 2020	Breast cancer	RCT/1.5/Multicenter	80	Fair
	Emens, 2020	Breast cancer	RCT/2/Multicenter	69	Good
	Cortés, 2022	Breast cancer	RCT/3/Multicenter	263	Good

*ALCL* anaplastic large cell lymphoma, *ALL* acute lymphoblastic leukemia, *AML* acute myeloid leukemia, *DLBCL* diffuse large B-cell lymphoma, *MF* mycosis fungoides, *NOS* Newcastle–Ottawa scale, *pcALCL* primary cutaneous ALCL, *PTCL* peripheral T cell lymphoma, *RCT* randomized controlled trial

**Table 3:**
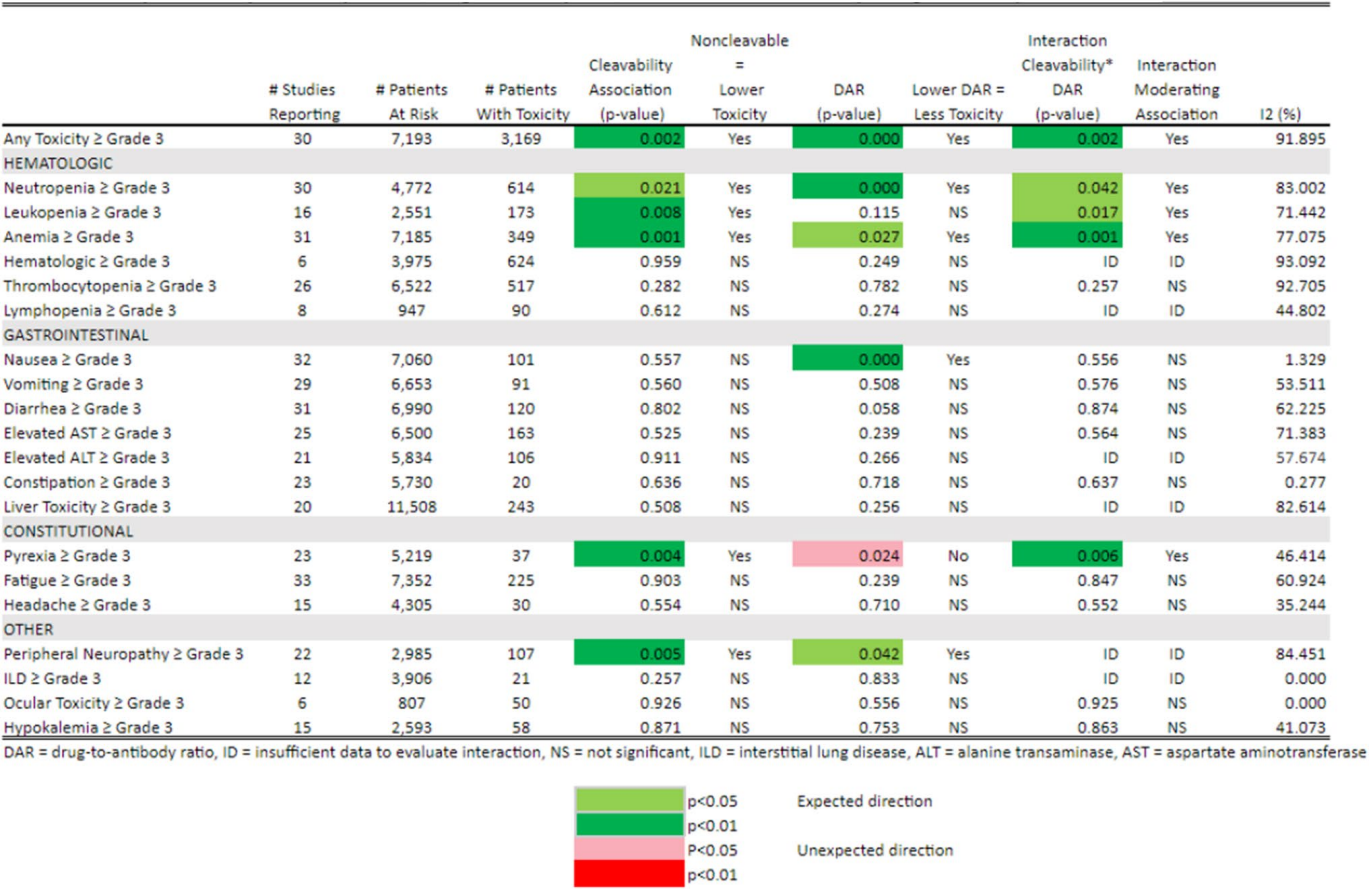
Toxicity ≥ Grade 3 by Cleavability of Linker, Drug-to-Antibody Ratio, and Interaction of Cleavability*Drug-to-Antibody Ratio

**Table 4:**
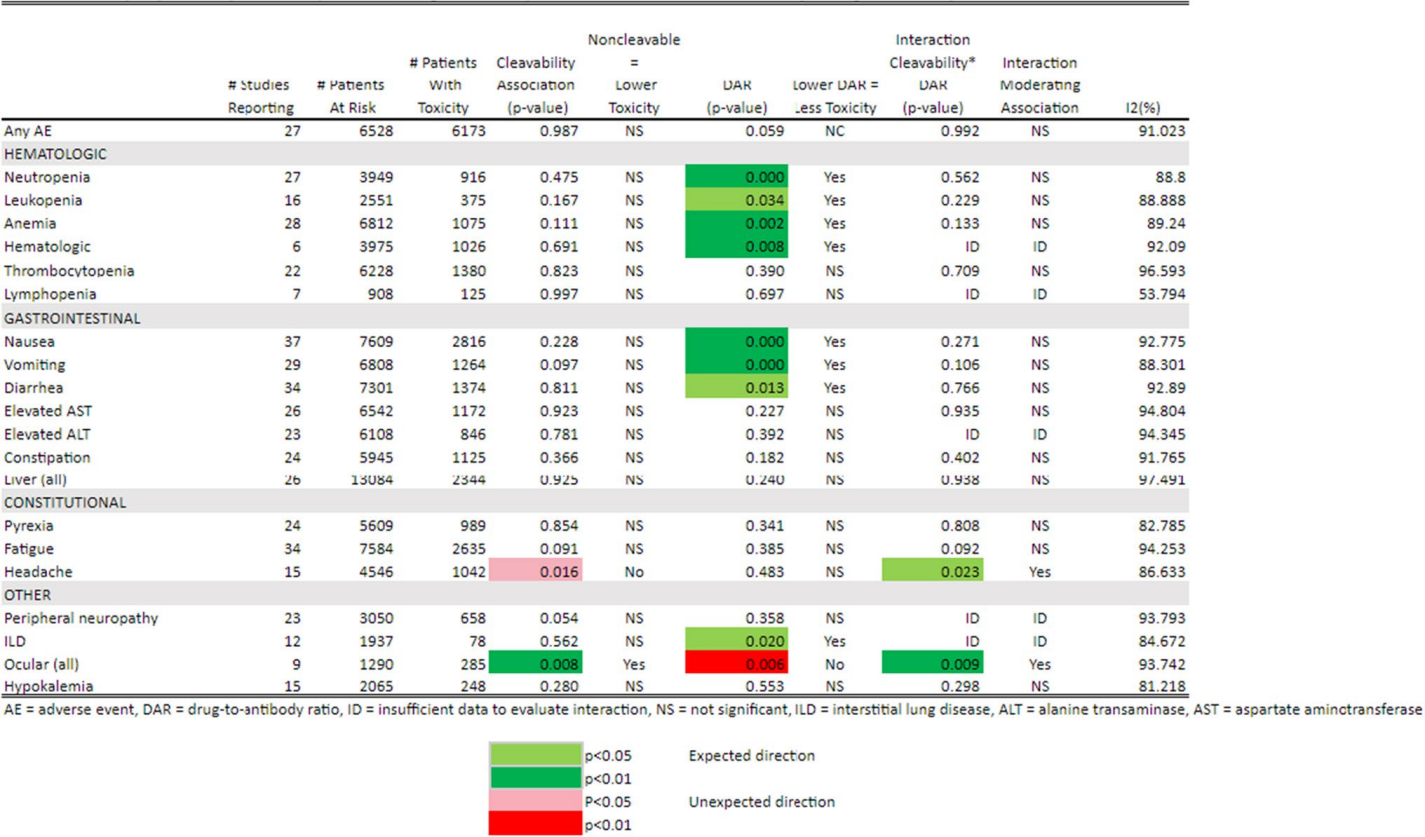
Toxicity Any Grade by Cleavability of Linker, Drug-to-Antibody Ratio, and Interaction of Cleavability * Drug-to-Antibody Ratio

As quantified in Tables 3 and 5, at least half the studies reported thrombocytopenia, neutropenia, anemia, increased AST and ALT, nausea, vomiting, diarrhea, and fatigue, as well as any toxicity at any grade and grade ≥ 3 AEs. Other toxicities were reported less frequently.

**Table 5:**
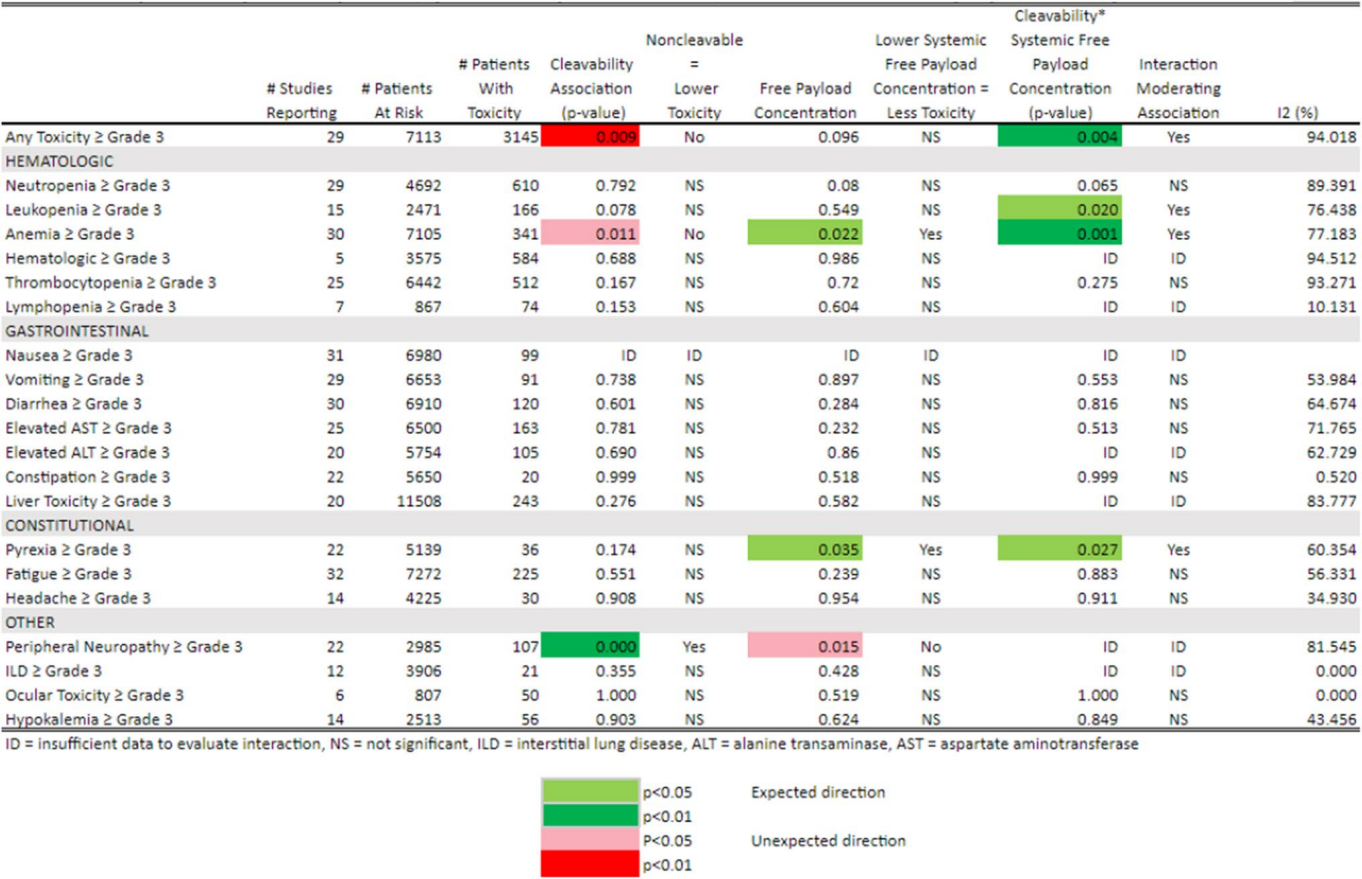
Toxicity ≥ Grade 3 by Cleavability of Linker, Systemic Free Payload Concentration, and Interaction of Cleavability * Systemic Free Payload Concentration

### Systemic toxicity rates with ADCs, stratified by cleavable vs non-cleavable linker

**Fig. 2:**
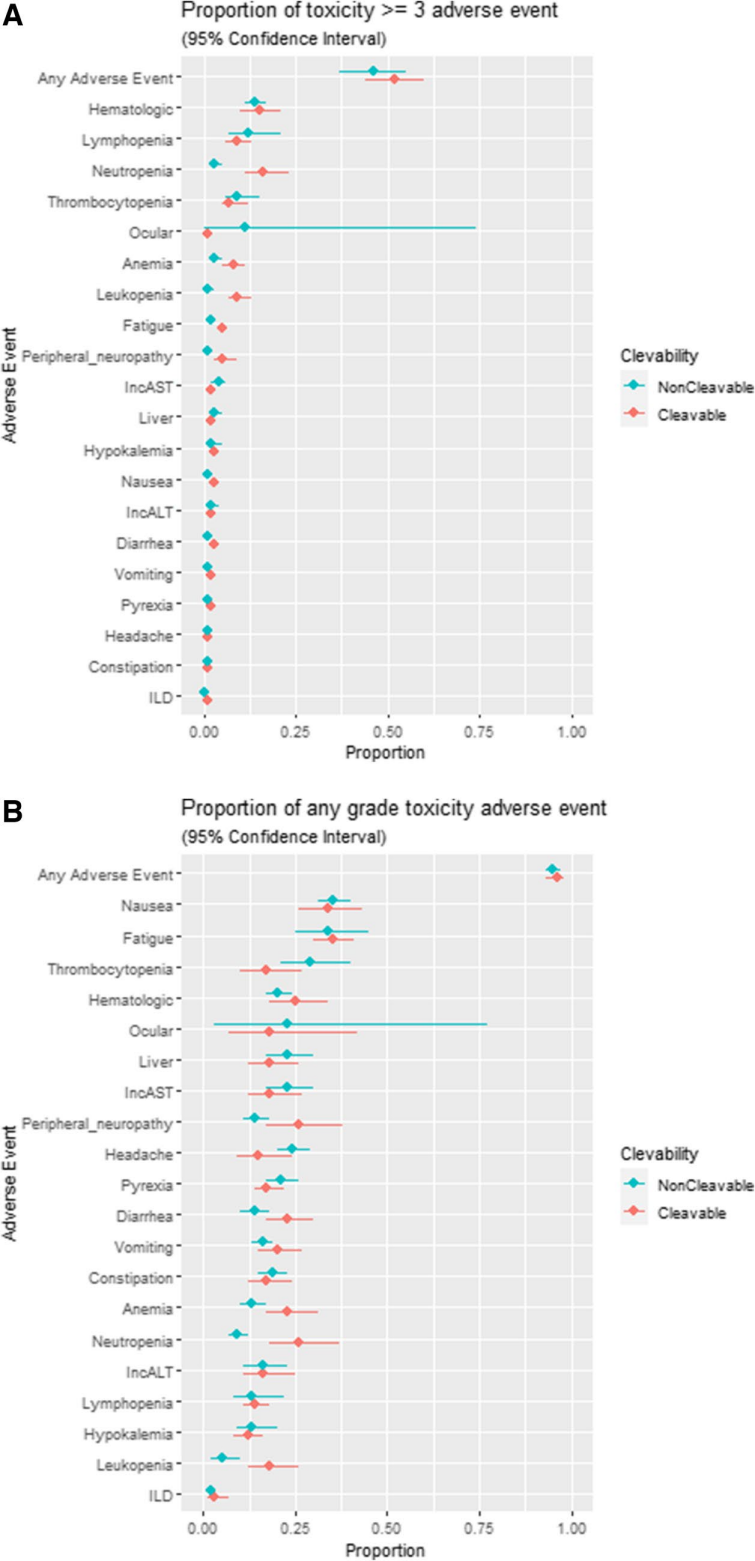
A. Proportion of patients experiencing each of the 21 toxicities ≥ grade 3 with 95% confidence interval. ILD = interstitial lung disease, IncAST = increased aspartate aminotransferase, IncALT = increased alanine aminotransferase. **B**: Proportion of patients experiencing each of the 21 toxicities any grade with 95% confidence interval. ILD = interstitial lung disease, IncAST = increased aspartate aminotransferase, IncALT = increased alanine aminotransferase

The meta-analytic point estimate of the proportion of patients experiencing each of the 21 toxicities with a 95% confidence interval is displayed in Fig. 2A for toxicities ≥ grade 3, and in Fig. 2B for any grade toxicity, both stratified by linker type. Composite AEs ≥ grade 3 occurred in 43% of patients overall, 47% in the cleavable linker-treated patients and 34% in the non-cleavable-treated patients, and these differences were significant (weighted risk difference −12.9%; 95% CI: −17.1% to −8.8%). Specific toxicities ≥ grade 3 with significantly lower proportions favoring non-cleavable linkers were neutropenia (−9.1%; 95% CI −12% to −6.2%) and anemia (−1.7%; 95% CI −3.3% to −0.1%). There was no significant difference in rates of grade ≥ 3 thrombocytopenia, increased AST/ALT, or fatigue. For all grade toxicities, there were no significant differences in rates of nausea, vomiting, diarrhea, hypokalemia, or headache.

### Linker type and drug-to-antibody ratio

We further examined the potential association between ADC linker type and drug-to-antibody ratios and the estimated probabilities of systemic toxicity. Since the linker type and drug-to-antibody ratio are design features of each ADC and thus are not independent factors, the interaction between linker type and drug-to-antibody ratio was modeled and estimated whenever feasible. A summary of the results for the 21 toxicities are represented as a heatmap (Tables 3 and 4). The p-values are color-coded for level of significance and direction of association.

For grade ≥ 3 toxicities (Table 3), non-cleavable linkers remain significantly and independently associated with lower toxicity for any AE (*p* = 0.002), neutropenia (*p* = 0.021), leukopenia (*p* = 0.008), anemia (*p* = 0.001), pyrexia (*p* = 0.004), and peripheral neuropathy (*p* = 0.005) when adjusted for DAR and their interaction where estimable. In addition, higher DAR was significantly and independently associated with higher probability of grade ≥ 3 toxicity for any AE, neutropenia, anemia, nausea (p < 0.001), and peripheral neuropathy. Higher DAR was significantly and independently associated with lower probability of grade ≥ 3 pyrexia. There were significant interaction terms between cleavability status and DAR for any AE (*p* = 0.002), neutropenia (*p* = 0.042), leukopenia (*p* = 0.017), anemia (*p* = 0.001), and pyrexia (*p* = 0.006). These were all moderating interactions, indicating a lower toxicity then would be predicted from the additive effects of a linear increase in DAR and linker cleavability type.

For any grade toxicity (Table 4), non-cleavable linkers were not significantly and independently associated with toxicity adjusted for DAR and their interaction except lower ocular and higher headache toxicity of any grade. However, higher DARs were significantly and independently associated with a higher probability of any grade toxicity for neutropenia, anemia, leukopenia, all hematological toxicities, nausea, vomiting, diarrhea, and interstitial lung disease. Although the sample size is limited, higher DAR was significantly and independently associated with lower probability of any grade ocular toxicity.

As observed in the heatmap of these data, the direction of the associations when significant were typically in the pre-specified clinically expected direction: higher DAR was associated with higher probability of grade ≥ 3 toxicity. As reported in Tables 3 and 4, most of the I [2] statistics are > 50% indicating at least moderate to high levels of heterogeneity in reported probabilities of each specific toxicity between studies.

### Linker type, systemic free payload agent concentration, and toxicity

Next we considered the potential association between ADC linker type and the systemic free payload concentration, including the potential interaction between linker type and systemic free payload concentration when estimable. These results are summarized as a heatmap of the p-values for the significance of the estimated coefficients for each factor in Tables 5 (toxicities ≥ grade 3) and 6 (any grade toxicity). The p-values are color-coded for level of significance and direction of regression coefficient associations between systemic free payload concentrations and linker type. For grade ≥ 3 toxicities (Table 5), non-cleavable linkers remain significantly and independently associated with lower toxicity for only peripheral neuropathy (*p* = 0.001). Neutropenia, leukopenia, and pyrexia no longer had a significant independent association with linker type after adjustment for the systemic free payload concentration. In fact, the probability of any AE ≥ grade 3 (*p* = 0.009) and anemia (*p* = 0.011) was higher in patients treated with non-cleavable linkers when adjusted for systemic free payload concentrations. Similarly, higher systemic free payload concentrations were significantly and independently associated with higher probability of anemia (*p* = 0.022) and pyrexia (*p* = 0.035). For any grade toxicities (Table 6), non-cleavable linkers remain independently associated with lower peripheral neuropathy (*p* = 0.025) but no other toxicity when adjusted for systemic free payload concentration. The main effect of higher systemic free payload concentration was significantly associated with increased probability for any toxicity (*p* = 0.043), anemia (*p* = 0.028), lymphopenia (*p* = 0.005), nausea (p < 0.001), vomiting (*p* = 0.006), constipation (*p* = 0.041), and interstitial lung disease (*p* < 0.001) after adjustment for linker type.

Again the I[2] statistics tend to be > 50% indicating at least moderate to high levels of heterogeneity in probabilities of the toxicities between studies.

**Table 6:**
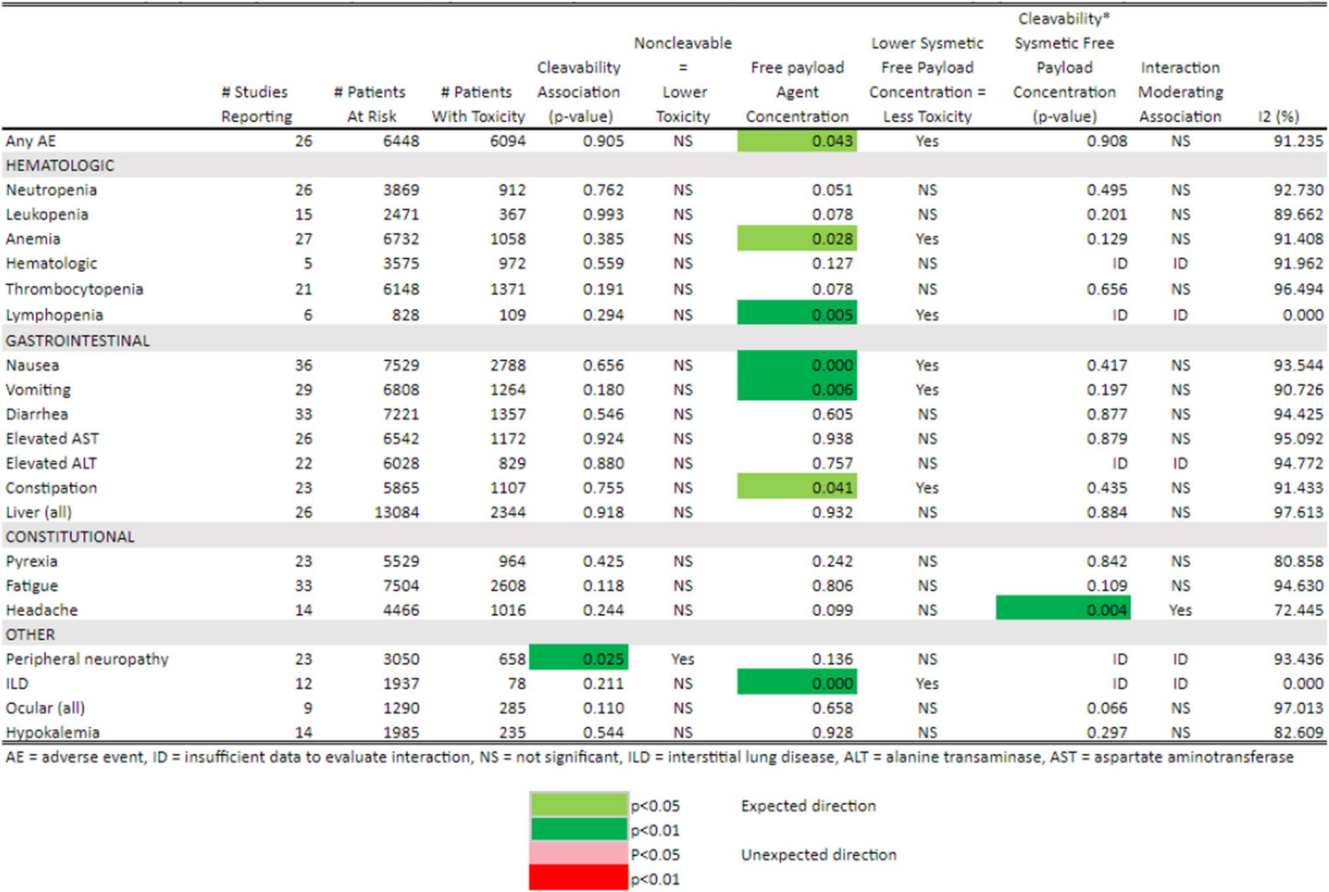
Toxicity Any Grade by Cleavability of Linker, Systemic Free Payload Concentration, and interaction of Cleavability * Systemic Free Payload Concentration

### ADC linker type, DAR, and free payload concentration

**Fig. 3:**
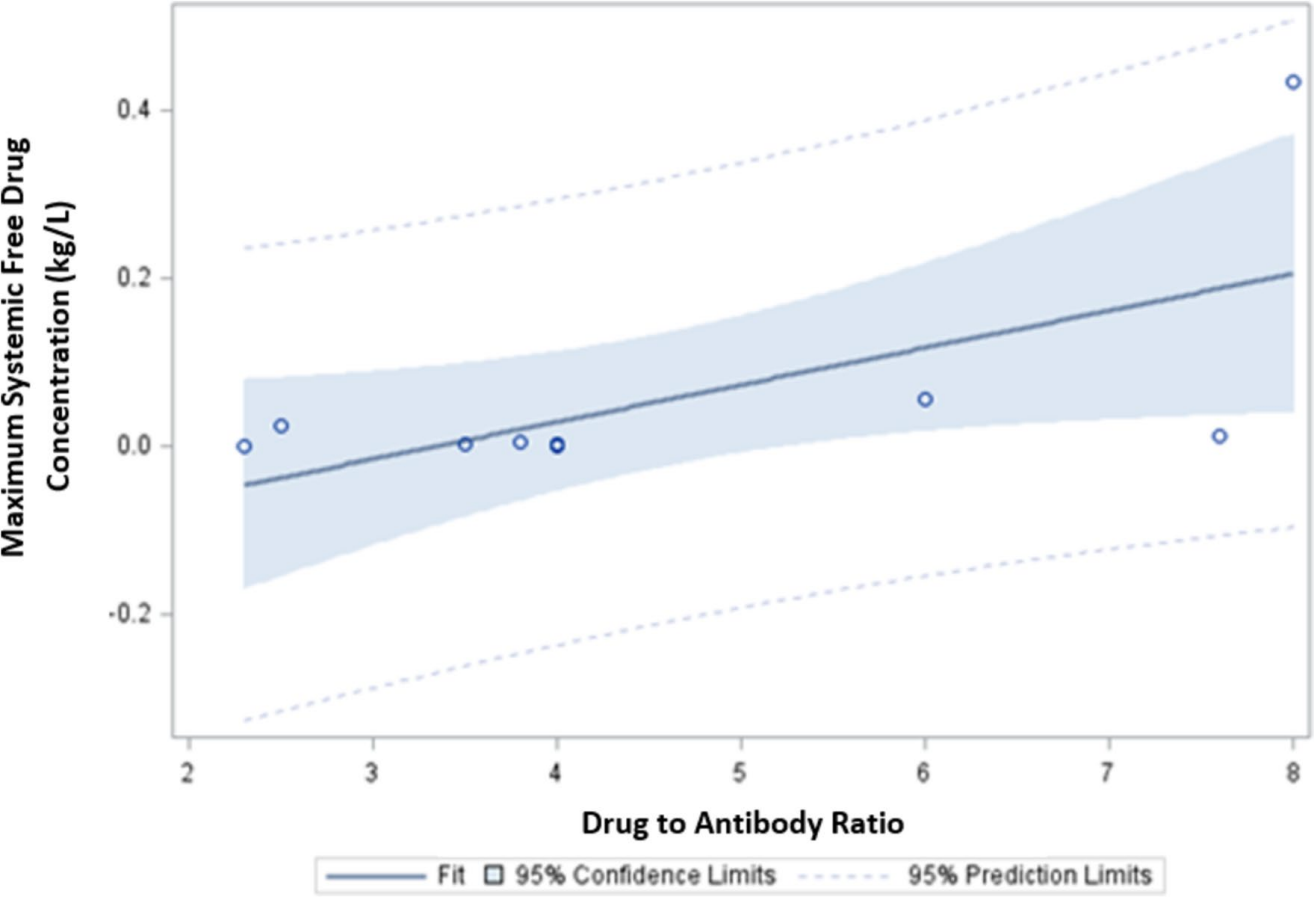
Association between DAR and maximum systemic free payload concentration

 As noted, linker-type, DAR, and systemic free payload concentration are not necessarily independent varying characteristics for each ADC. To further investigate this, we described the relationships between cleavability type, DAR, and measured systemic free payload concentration for the 11 FDA-approved ADCs under evaluation in this study. These characteristics are summarized in Table 1. Non-cleavable linkers have a numerically lower mean estimated DAR (3.75 ± 0.35 versus 4.78 ± 2.18, *p* = *0.544*) and lower estimated systemic free payload concentration (1.89 × 10^–5^ m^2^/L ± 1.41 × 10^–5^ m^2^/L versus 1.62 × 10^–3^ m^2^/L ± 3.64 × 10^–3^ m^2^/L, *p* = *0.567*). These differences are not statistically different, likely due to the small number of agents being compared (2 non-cleavable and 9-cleavable). However, the higher measured systemic free payload concentrations were significantly associated with higher DARs (*p* = 0.043) as depicted in Fig. 3.

## Discussion

In this review and meta-analysis, we sought to delineate how features of ADC design, including linker cleavability, DAR, and systemic free payload concentration, may contribute to their associated systemic toxicities. The results support the hypothesis that ADCs with cleavable linkers are associated with more systemic toxicities than those with non-cleavable linkers. Interestingly, even though higher DAR was associated with higher grade ≥ 3 toxicity, the apparent protective effect of the non-cleavable linker persisted even after adjusting for DAR. However, we found that the association between non-cleavable linkers and lower toxicity was *not* observed after adjusting for systemic free payload concentration. This suggests that systemic free payload concentration is the main factor driving toxicity in ADC-treated patients.

Notably, trastuzumab deruxtecan (T-DXd) has a considerably higher systemic free payload concentration than the other agents studied here. T-DXd has a tetrapeptide cleavable linker that may make it more vulnerable to premature release of its payload. Such a prematurely released payload might explain why T-DXd may be effective in tumor control regardless of HER2 expression. Emerging clinical evidence supports this hypothesis. In a small trial of T-DXd in non-small cell lung cancer patients, the activity of T-DXd was shown to be independent of HER2 over-expression in HER3 + , 2 + , or 1 + tumors [47]. More recently, clinical trial data presented during the 2021 San Antonio Breast Cancer Symposium showed that T-DXd was active in breast cancer patients regardless of HER2 expression, including HER2 0 tumors [48].

By contrast, trastuzumab emtansine (T-DM1), another anti-HER2 ADC with a non-cleavable linker, has not been shown to have activity in patients with low HER2 tumor expression. This may partly explain why in the DESTINY-03 trial[35], T-DM1 (HER2 dependent) was shown to have lower efficacy compared to T-DXd (HER2 dependent and independent). Consonant with these observations, T-DM1 was also shown to have much lower systemic toxicity compared to T-DXd, likely because of the relative stability of the T-DM1 linker. We’ve previously proposed that the off-target effects observed with T-DM1 may be the result of payload released from lysed tumor cells [2]. It is tempting to speculate that for these reasons, ADCs with cleavable linkers may in general have higher anti-tumor efficacy albeit higher systemic toxicities.

Our results arise from a meta-analysis using multiple agents used to treat highly disparate patient populations with different malignancies. We suspect this explains most of the heterogeneity observed in probabilities of specific toxicities between studies reflected in the I [2] statistics. Nonetheless, differences in ADC chemical design are shown to be significantly associated with specific clinical toxicities despite this tremendous heterogeneity.

## Conclusion

In summary, ADCs are rapidly becoming the standard of care for patients across disease sites. The results here show that linker choice and the potential for premature payload release among ADCs can affect their systemic toxicity and efficacy. It will, therefore, be critical in the design of future ADCs to find the appropriate balance between the highest potential efficacy and associated systemic toxicities [49]. In this regard, contemporary studies are focused on the development of novel ADC linkers that can be released conditionally within the tumor microenvironment to increase both the specificity of drug delivery and anti-tumor efficacy. The ideal ADC design should aim for high therapeutic index that balances off-target toxicities, taking into consideration factors such as linker cleavability, DAR, and payload membrane permeability [50]. The results presented here suggest one critical consideration in achieving this balance during future ADC development for cancer patients will be linker design and the potential for premature payload release.
